# How Following Medical Artificial Intelligence Advice Can Mitigate Malpractice Liability: Cross-National Insights from a Randomized Trial

**DOI:** 10.2967/jnumed.126.272292

**Published:** 2026-09

**Authors:** Alessandro Tacconelli, Jakob Merane, Aileen Nielsen, Kevin Tobia, Björn Hackanson, Alexander Stremitzer

**Affiliations:** 1 Center for Law and Economics, ETH Zurich, Zurich, Switzerland;; 2 School of Law, Harvard University, Boston, Massachusetts;; 3 School of Law, Georgetown University Law Center, Washington, DC; and; 4 Comprehensive Cancer Center Augsburg as Part of the CCC-WERA Consortium, University Medical Center Augsburg and Department of Medicine I, University Faculty of Medicine, Medical Center Freiburg, University of Freiburg, Germany.; † Current address as of July 1, 2026: The School of Law, University of Massachusetts-Dartmouth, Dartmouth, Massachusetts.

**Keywords:** artificial intelligence, liability, precision medicine, legal systems

## Abstract

Artificial intelligence (AI) increasingly influences clinical decision-making, yet its recommendations may diverge from standard care. Although malpractice concerns are thought to discourage physicians from following AI advice, experimental evidence from the United States suggests the opposite: lay jurors are more likely to hold physicians liable when they reject AI recommendations. Whether this pattern extends to systems in which court-appointed experts, not lay jurors, determine liability remains unknown. **Methods:** To examine how physicians and laypeople in expert-based and lay-juror legal systems evaluate physicians’ acceptance or rejection of AI recommendations, particularly when those recommendations deviate from standard care, we designed a randomized vignette study: a 2 × 2 factorial design varying the AI recommendation (standard vs. nonstandard care) and a fictional physician’s decision (accept vs. reject). The study was conducted online in 2023 among nationally representative samples of U.S. and German adults and from 2023 to 2024 among German physicians. In total, 387 German physicians, 2291 U.S. adults, and 2283 German adults participated; those not completing the survey or failing attention checks were excluded per preregistered criteria. Participants were randomly assigned to 1 of 4 vignettes, varying the AI recommendation (standard vs. nonstandard care) and physician’s decision (accept vs. reject). The reasonableness of the fictional physician’s decision was measured, rated by participants on a Likert scale. **Results:** Analysis, following preregistered exclusion criteria, included 248 German physicians, 1202 U.S. adults, and 1358 German adults. Physicians accepting standard-care AI recommendations were rated more reasonable than those rejecting them (U.S. laypeople: *t* = 5.36; 95% CI, 0.45–0.97; *P* < 0.001; German physicians: *t* = 2.47; 95% CI, 0.14–1.30; *P* = 0.02; German laypeople: *t* = 4.14; 95% CI, 0.27–0.76; *P* < 0.001). Ratings of physicians accepting versus rejecting AI nonstandard-care recommendations were statistically equivalent. Equivalence was tested at an α-value of 0.05 using a two 1-sided tests procedure, reported with 90% CIs per standard convention (U.S. laypeople: *t* = –4.90; 90% CI, –0.1 to 0.36; *P* < 0.001; German physicians: *t* = –1.76; 90% CI, –0.12 to 0.67; *P* = 0.04; German laypeople: *t* = 5.35; 90% CI, –0.35 to 0.06; *P* < 0.001). **Conclusion:** Across the United States and Germany, samples representative of lay jurors and court-appointed experts viewed accepting standard-care AI advice as more reasonable, whereas accepting or rejecting nonstandard-care AI advice was judged similarly. Contrary to predictions, malpractice liability regimes do not necessarily pose a barrier to AI use in precision medicine.

## Visual Abstract

**Figure:**
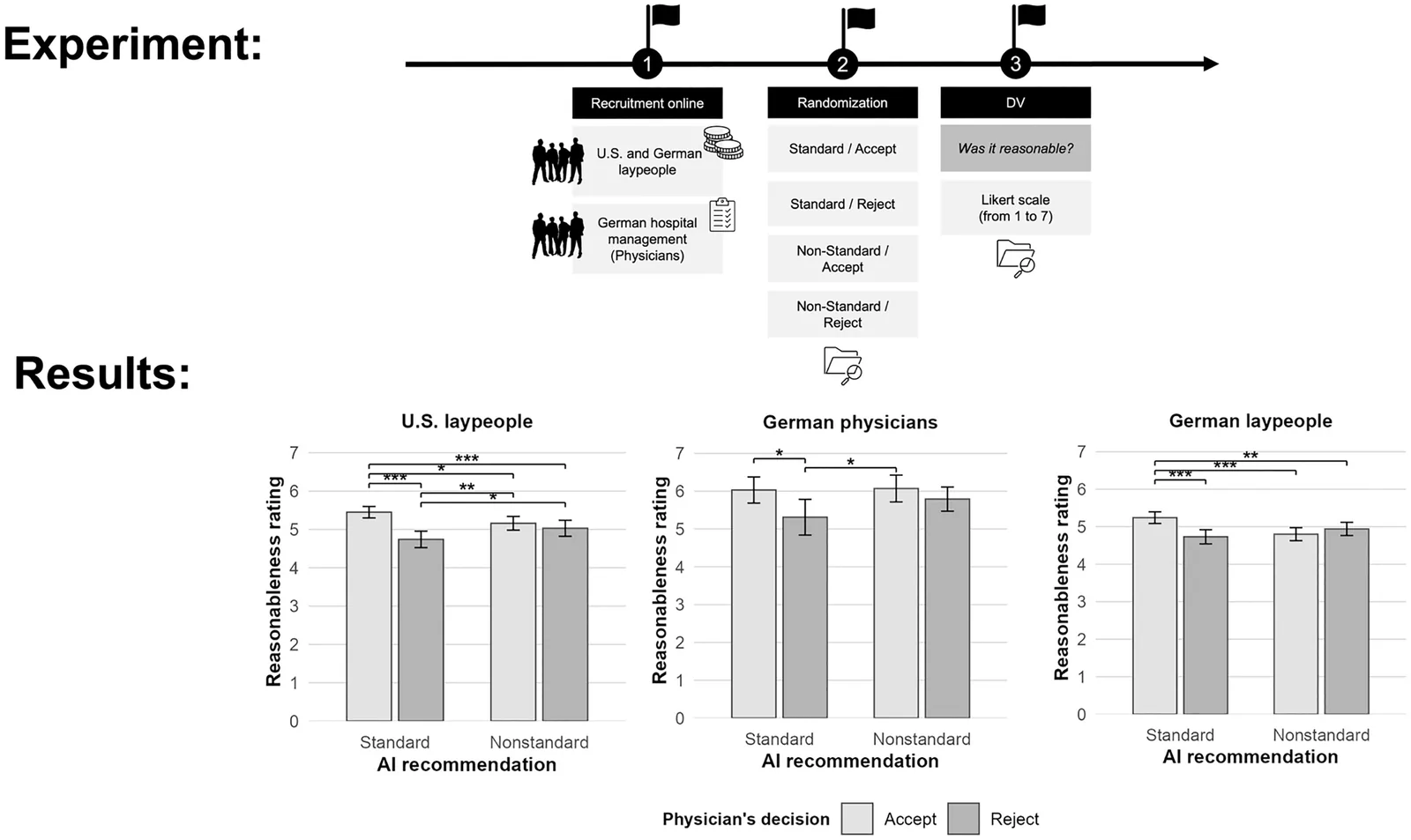

Artificial intelligence (AI) systems are increasingly integrated into clinical practice, offering personalized diagnostic and treatment recommendations that may reiterate conventional guidelines or diverge from them (1–3). Although these tools hold the potential to improve patient outcomes, their use raises novel questions regarding legal liability (4). Scholars have expressed concerns that existing tort frameworks may discourage physicians from following AI-generated recommendations (5).

Specifically, Price et al. (6) suggested that U.S. law might hinder the effective deployment of medical AI. Since tort law tends to favor adherence to the established standard of care, using AI as a source of personalized treatments that may deviate from standard protocols could increase physicians’ exposure to liability.

The experimental findings of Tobia et al. (7) challenged this view. In the United States, negligence is determined by lay juries who, after hearing testimony from dueling medical experts, ultimately evaluate the reasonableness of care provided (8–9). Hence, to investigate how jurors assess physician liability in cases involving AI-assisted decisions, Tobia et al. conducted a vignette study with jury-eligible U.S. adults. Physicians’ actions were judged to be more reasonable when they accepted, rather than rejected, AI recommendations that aligned with standard care. When AI recommended nonstandard care, there was no meaningful difference in reasonableness judgments between accepting and rejecting the advice. These results suggest that U.S. tort law may not impede, and may even facilitate, the adoption of AI tools in clinical care.

Yet these findings are limited in 2 respects. First, the study focused exclusively on the United States, where negligence is adjudicated by lay jurors. Thus, it remains unclear whether these findings generalize to jurisdictions in which court-appointed experts, rather than lay jurors, play the most central role in liability adjudications. Second, both AI technologies and public attitudes toward AI are evolving rapidly, and it is unknown whether the now 5-y-old findings about U.S. laypeople’s views of medical AI have evolved.

To address these limitations, we first replicated the study by Tobia et al. (7) to test for intertemporal consistency. Second, we expanded the investigation by including a sample of German physicians. This allowed us to compare the responses of U.S. laypeople, who could serve as potential jurors in the U.S. tort system, with those of German physicians, who serve as de facto decision-makers in systems such as Germany’s (9–11). We also recruited a sample of German laypeople as a control group to assess whether any differences in liability judgments could be attributable to cultural background alone or might be explained by physicians’ tendency to evaluate other physicians more favorably.

## MATERIALS AND METHODS

### Legal Framework for Medical Liability

Medical malpractice in both the United States and Germany is generally analyzed through a common doctrinal framework requiring proof of a duty of care, a breach of that duty, and a causal link between the breach and the patient’s injury (12).

In the United States, malpractice claims are typically adjudicated within an adversarial system in which the burden of proof rests with the plaintiff (12). Fact-finding is commonly entrusted to lay juries, which are asked to evaluate competing expert testimony presented by the parties. Because these experts are retained by each side, their opinions may reflect partisan positions, and jurors must determine which account more persuasively defines reasonable medical practice (9).

In Germany, by contrast, malpractice adjudication takes place in a system in which judges assume a more active role in the evaluation of evidence (10). Although the burden of proof likewise generally rests with the patient, courts routinely appoint independent medical experts whose opinions carry substantial, and often decisive, weight in determining whether the standard of care was breached (10–11). Experts retained by the parties are permitted, but they typically carry less evidentiary weight than court-appointed experts.

Across both systems, the concept of reasonableness has evolved from a model closely tied to customary professional practice toward a more flexible, context-sensitive standard centered on what a competent physician would do under the circumstances and in light of available knowledge and resources (8). This development is especially important in the context of AI, in which recommendations may diverge from established guidelines and clinical practice. Understanding how legal decision-makers assess the reasonableness of AI-assisted clinical decisions is therefore essential for anticipating the liability implications of emerging technologies.

### Models of Medical Liability

**FIGURE 1.**
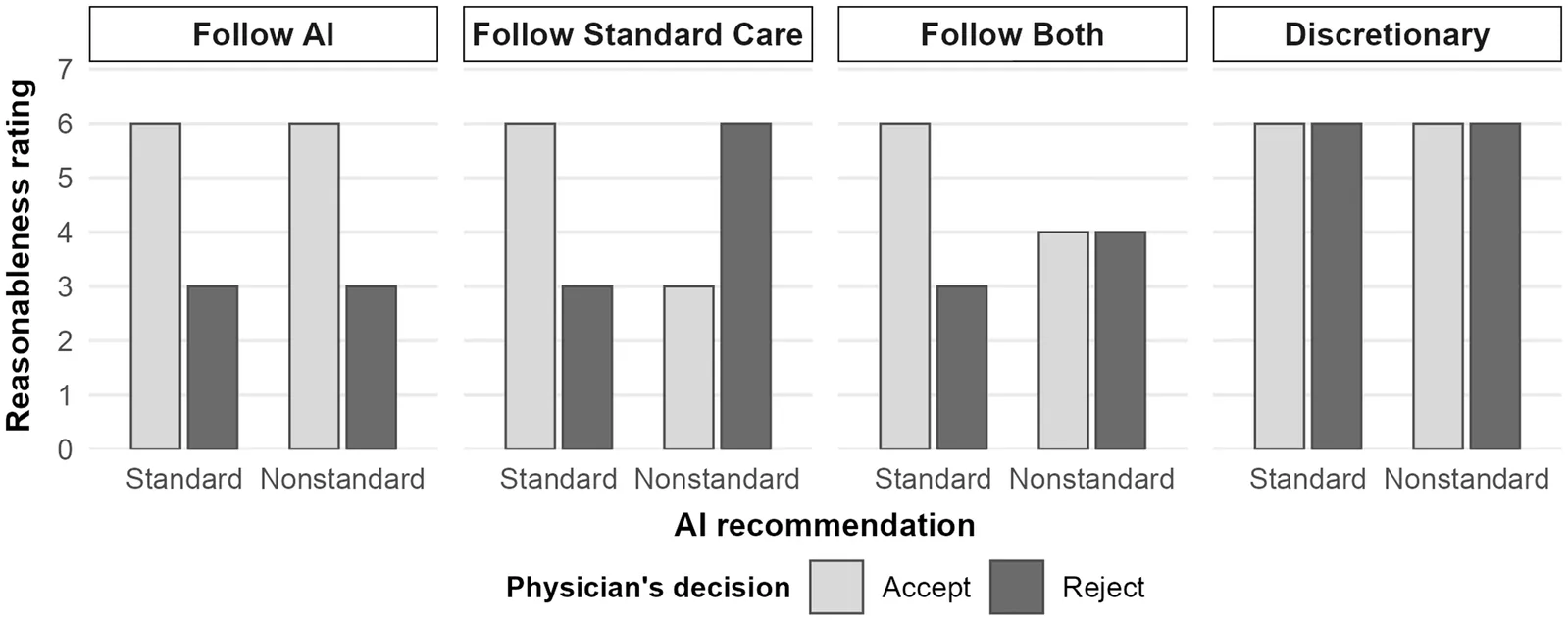
Predictive models of medical liability. This figure illustrates experimental predictions of 4 liability models. *x*-axis displays experimental condition for each model. *y*-axis shows expected reasonableness ratings, on 7-point Likert scale (1 = very unreasonable, 7 = very reasonable).

To evaluate our findings, we applied the 4 liability models proposed by Tobia et al. (7), which build on the framework of Price et al. (6). These models (Fig. 1) offer different predictions about how reasonable a physician’s decision appears (and, thus, the likelihood of liability), based on 2 factors: the nature of the care provided (standard vs. nonstandard) and the physician’s response to AI recommendations (accept vs. reject).

In the Follow AI Model, physicians who follow AI recommendations are shielded from liability, regardless of whether the advice aligns with standard care. Conversely, following AI advice reduces the risk of liability. Under the Follow Standard Care Model, liability is determined by adherence to standard care, regardless of which recommendation originates from AI. Thus, deviating from standard care increases liability risk. The Follow Both Model posits an interaction between the type of care and the physician’s decision: accepting standard-care AI recommendations reduces liability risk, whereas rejecting them increases it. When AI recommends nonstandard care, physicians face conflicting risks, leaving no clear legal preference. The Discretionary Model assumes that neither standardness of care nor adherence to AI plays a significant role in liability judgments. All combinations of factors produce similar liability assessments, reflecting a deferential approach.

### Hypotheses

We used these models as predictive frameworks for our investigation.

In our replication, we tested the primary hypothesis that the Follow Both Model captures judgments of physicians’ decisions. In the extension involving German physicians, we adopted an exploratory approach: anticipating condition-dependent differences across experimental groups but without committing to any specific model a priori. The same exploratory approach was applied to the German laypeople’s sample.

### Experimental Design

**FIGURE 2.**
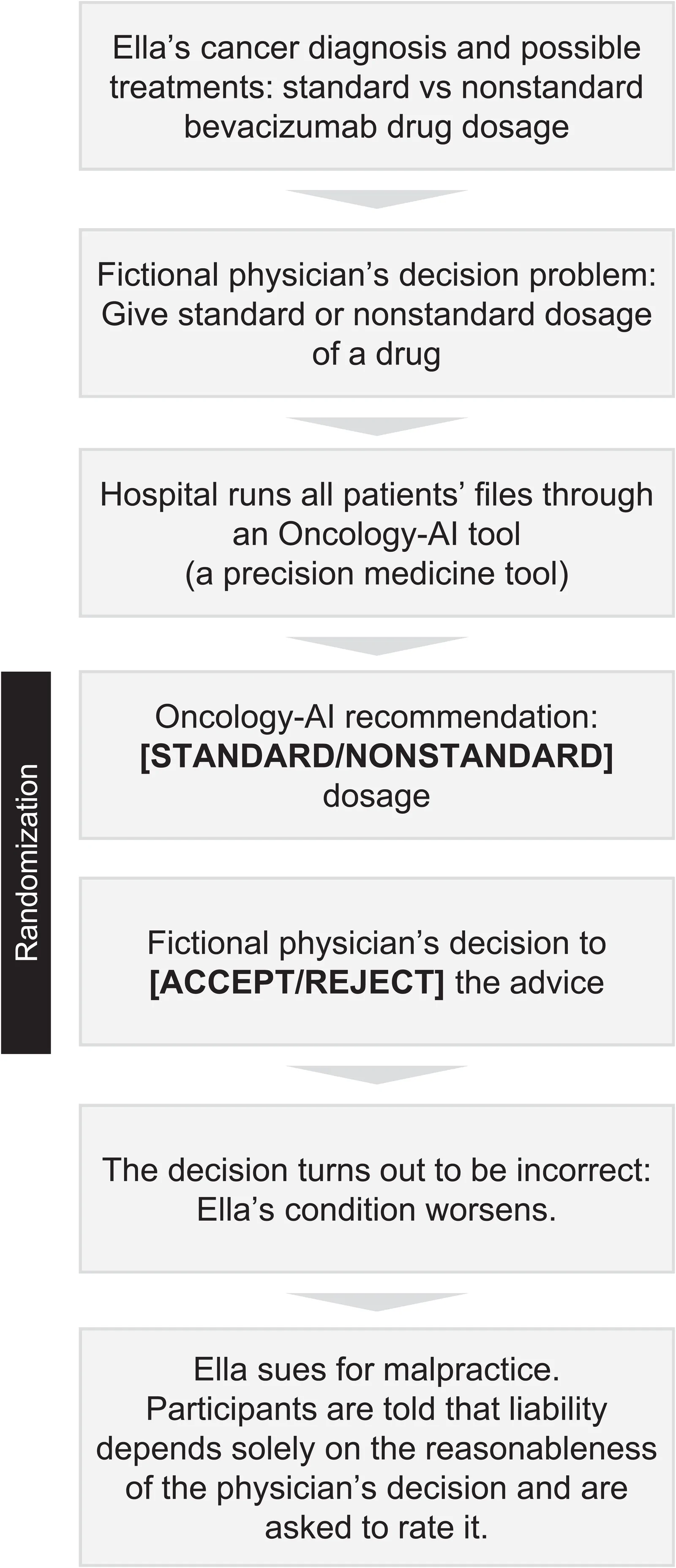
Flowchart of vignettes.

All participants across our 3 samples were recruited online and completed a vignette survey on the Qualtrics platform. To limit researcher degrees of freedom, we used the scenario originally suggested by Price et al. (6) and tested by Tobia et al. (7). The scenario described the case of Ella, a patient with ovarian cancer harmed after receiving either a standard or a nonstandard chemotherapy dosage from a fictional physician after that physician had consulted the hospital’s default AI system (Fig. 2). The AI tool was presented as an institutionally implemented, routinely used decision-support system rather than as a novel or experimental technology.

We used a 2 × 2 between-participant design, resulting in 4 experimental vignettes. The vignettes varied along 2 dimensions: the type of treatment recommended by the AI tool (standard vs. nonstandard) and the physician’s decision (accept vs. reject the recommendation).

Participants were randomly assigned to a single vignette. In all conditions, the occurrence of patient harm was held constant. The inclusion of an adverse outcome was necessary to situate the scenario within a malpractice framework, in which liability assessments presuppose injury. By fixing the outcome across conditions, the design isolates the effect of the physician’s decision and the AI recommendation on participants’ evaluations.

After reading the physician’s decision, the participants rated its reasonableness and answered questions on demographics, attitudes toward AI, and exposure to the legal and medical fields.

### Outcome Variable

Our main outcome was participants’ stated reasonableness of the physician’s decision. Using a 7-point Likert scale, participants rated their agreement (1 = strongly disagree, 7 = strongly agree), with the following statement: “Dr. Huber’s [or Dr. Jones’ for U.S. participants] treatment decision, including the [acceptance/rejection] of Oncology-AI’s recommendation to provide the [standard/nonstandard] dosage, was one that could have been made by other reasonably careful medical practitioners in the same or similar circumstances.”

To anchor this assessment in a legal context, participants were instructed to evaluate the scenario as part of a malpractice case. Specifically, they were asked to imagine that the patient had initiated a lawsuit against the physician and that they had been assigned the role of a decision-maker (juror for lay participants; medical expert for physicians). Participants were further informed that causation was not in dispute (i.e., the physician’s treatment decision had led to the patient’s deterioration) and that the only central question left to decide for liability was whether the physician’s conduct was consistent with what reasonably careful practitioners would have done under similar circumstances.

### Statistical Analysis

The reasonableness ratings of the fictional physician’s decisions were compared across the 4 experimental groups using 2 × 2 ANOVA and post hoc pairwise *t* test comparisons. In cases of null results, two 1-sided tests (TOST) procedures were performed to check whether the null hypothesis was the most plausible explanation, with equivalence bounds set at a medium effect size of a Cohen d of 0.5 as preregistered and results reported with 90% CIs per standard convention (13). Robustness checks were conducted using multivariate regression models that included demographic variables, attitudes toward AI, and prior exposure to the legal or medical fields as covariates, as these factors could influence malpractice judgments. Adjusting for them allowed assessment of whether the primary effects were independent of these background characteristics.

In addition, we conducted a pooled analysis across all 3 samples to examine overall differences in reasonableness judgments. Comparisons between U.S. laypeople and German physicians were used to explore potential cultural influences, whereas comparisons between German laypeople and physicians assessed the role of selection and professional socialization. Regression models were used to evaluate differences across samples in the pooled dataset.

All analyses were performed with R software, version 4.4.2 (R Project for Statistical Computing). Except for the TOST procedure, which requires the execution of TOST, all tests were 2-sided and *P* values of 0.05 or less were considered statistically significant.

## RESULTS

**FIGURE 3.**
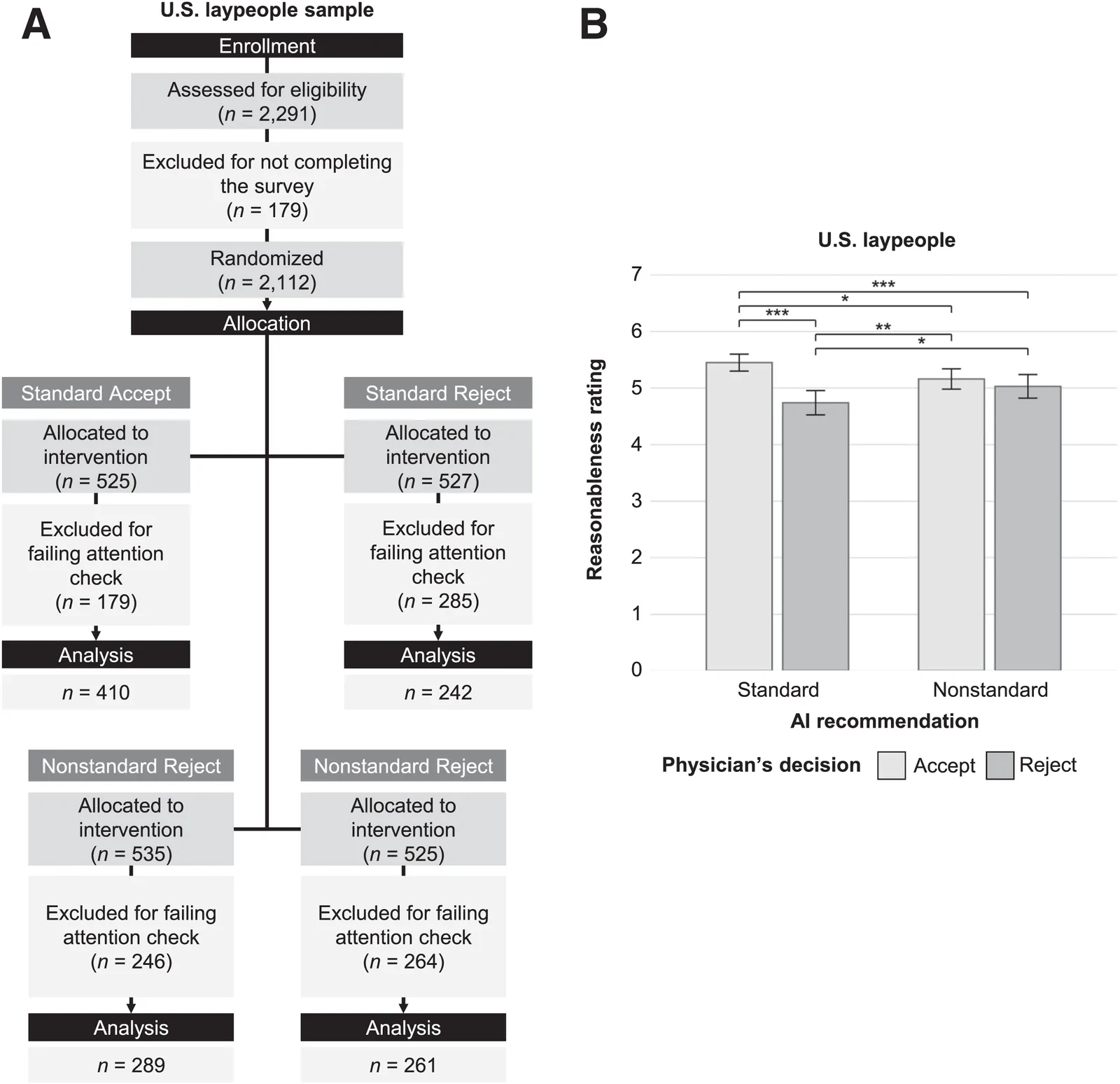
(A) CONSORT diagram: recruitment, randomization, and exclusion for U.S. laypeople. (B) Mean reasonableness ratings by recommendation and decision type for U.S. laypeople. Bars represent mean reasonableness ratings on 7-point scale. Error bars indicate 95% CI. *P ≤ 0.05. **P ≤ 0.01. ***P ≤ 0.001.

**FIGURE 4.**
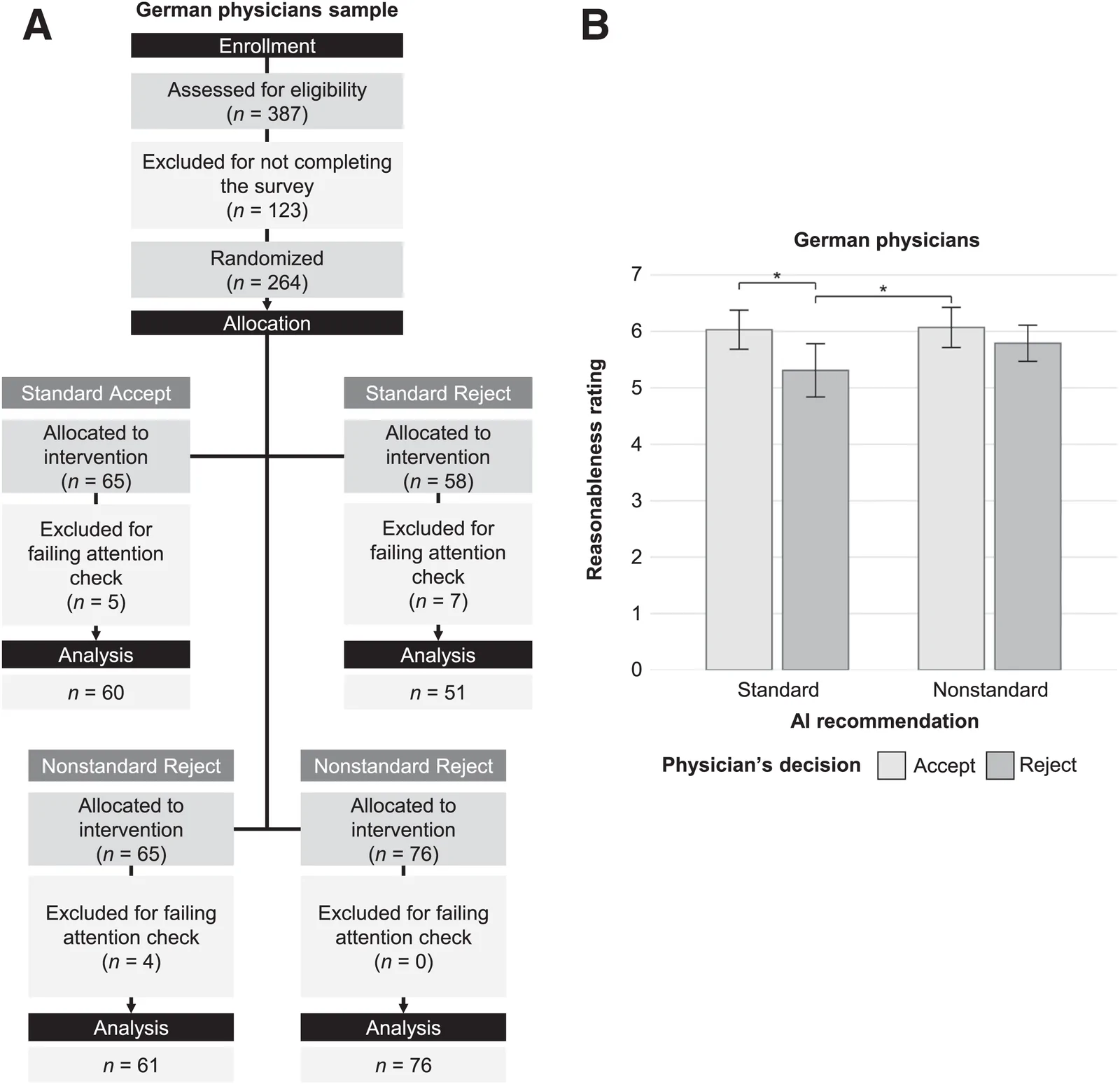
(A) CONSORT diagram: recruitment, randomization, and exclusion for German physicians. (B) Mean reasonableness ratings by recommendation and decision type for German physicians. Bars represent mean reasonableness ratings on 7-point scale. Error bars indicate 95% CI. *P ≤ 0.05.

**FIGURE 5.**
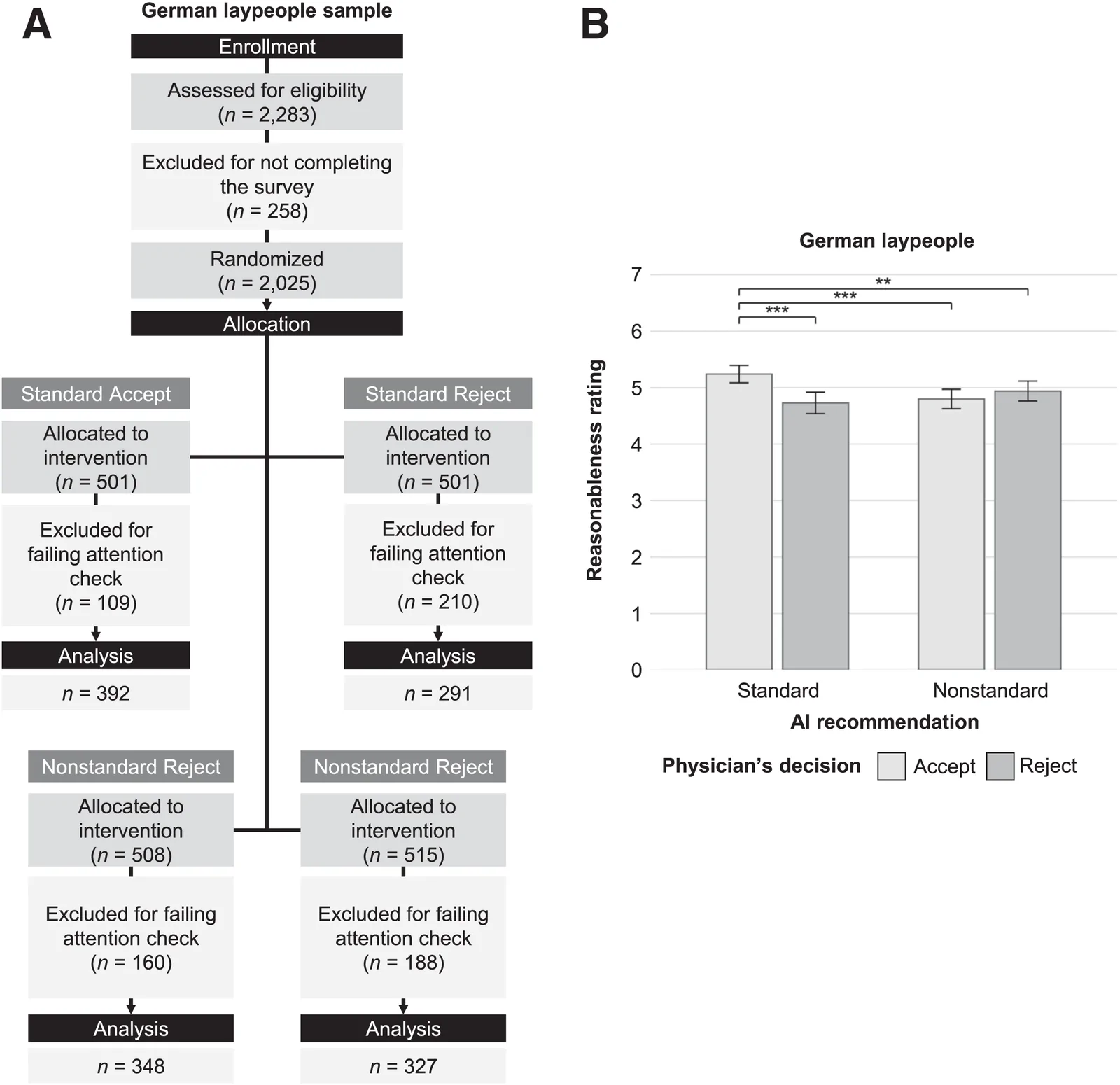
(A) CONSORT diagram: recruitment, randomization, and exclusion for German laypeople. (B) Mean reasonableness ratings by recommendation and decision type for German laypeople. Bars represent mean reasonableness ratings on 7-point scale. Error bars indicate 95% CI. **P ≤ 0.01; ***P ≤ 0.001.

In total, 2808 individuals, recruited between 2023 and 2024, were included in the final analysis after application of preregistered exclusion criteria: 1202 U.S. laypeople, 248 German physicians, and 1358 German laypeople (Consolidated Standards of Reporting Trials [CONSORT] flows are provided in Figs. 3–5). The project was approved by ETH Ethics Commission (approval EK 2021-N-221). All participants signed an informed consent form.

Baseline demographics for each sample, including age, sex, and race, are provided in Supplemental Tables 1–3 (supplemental materials are available at http://jnm.snmjournals.org) (14–21). No additional missing data occurred; all included participants provided complete responses to the survey. For the lay samples, a power analysis conducted with G*Power indicated that 1200 participants would be sufficient to detect small effects (Cohen d = 0.2) with 80% power. Recruiting physicians, a harder-to-reach population, posed more challenges. Accordingly, we preregistered only a general plan to include them. Although the final physician sample did not reach the target size, it was sufficient to detect statistically significant effects.

Lay participants were recruited through Cint, a commercial panel aggregation platform. For the recruitment, quotas were set to reflect the national populations of the United States and Germany based on sex, age, education level, income, and employment status. Participants received directly through Cint a standard panel compensation consistent with market rates for surveys of similar length. German physicians were recruited separately from 4 academic hospitals: University Hospital Augsburg, University Hospital of Cologne, Charité–Universitätsmedizin Berlin, and University Medical Center Mainz. Physicians did not receive financial compensation. Their participation was motivated by professional interest: they were informed that they would receive access to the study results on completion.

Pairwise *t* test results are reported in Table 1. To account for the multiplicity of hypothesis testing and control the probability of error, we used Holm *P* value adjustments (the adjusted *P* values are reported in the supplemental materials). All results held robust when controlled for demographic variables and an extended set of additional covariates that might be expected to influence malpractice judgments (Supplemental Tables 4–6).

**TABLE 1.** Pairwise *t* Tests for Each Sample

Parameter	Standard–reject	Nonstandard–accept	Nonstandard–reject
U.S. laypeople			
Standard–accept	*t* = 5.36, *P* < 0.001 [0.45, 0.97]	*t* = 2.41, *P* = 0.02 [0.05, 0.52]	*t* = 3.21, *P* = 0.001 [0.16, 0.67]
Standard–reject	NA	*t* = −2.99, *P* = 0.003 [−0.71, −0.15]	*t* = −1.94, *P* = 0.05 [−0.59, 0]
Nonstandard–accept	NA	NA	*t* = 0.94, *P* = 0.35 [−0.14, 0.41]
German physicians			
Standard–accept	*t* = 2.47, *P* = 0.02 [0.14, 1.30]	*t* = −0.13, *P* = 0.90 [−0.52, 0.46]	*t* = 1.03, *P* = 0.30 [−0.22, 0.71]
Standard–reject	NA	*t* = −2.55, *P* = 0.01 [−1.34, −0.17]	*t* = 1.67, *P* = 0.10 [−1.04, −0.09]
Nonstandard–accept	NA	NA	*t* = 1.15, *P* = 0.25 [−0.20, 0.75]
German laypeople			
Standard–accept	*t* = 4.14, *P* < 0.001 [0.27, 0.76]	*t* = 3.71, *P* < 0.001 [0.21, 0.67]	*t* = 2.47, *P* = 0.01 [0.06, 0.53]
Standard–reject	NA	*t* = −0.59, *P* = 0.56 [−0.33, 0.18]	*t* = −1.67, *P* = 0.09 [−0.48, 0.04]
Nonstandard–accept	NA	NA	*t* = −1.14, *P* = 0.25 [−0.39, 0.10]

NA = not applicable.

Values in brackets represent 95% CI. Nominal *P* values are reported (corresponding adjusted *P* values are reported in supplemental materials, where we also detail why interpretation of results remains consistent when accounting for these adjustments).

### U.S. Laypeople

The results for the U.S. laypeople (46.75% female) replicated the findings of Tobia et al. (7). A 2 (recommendation: standard vs. nonstandard) × 2 (decision: accept vs. reject) ANOVA detected a main effect of decision (accept vs. reject; *F*(1, 1198) = 21.29, *P* < 0.001) and a significant recommendation × decision interaction (*F*(1, 1198) = 9.36, *P* = 0.002). Yet, there was no significant effect of recommendation (standard vs. nonstandard; *F*(1, 1198) = 0.81, *P* = 0.37).

Mean reasonableness ratings were the highest in the standard–accept condition (mean, 5.45; SD, 1.54), followed by nonstandard–accept (mean, 5.16; SD, 1.55), nonstandard–reject (mean, 5.03; SD, 1.71), and standard–reject (mean, 4.74; SD, 1.70) (Fig. 3).

The ANOVA and pairwise *t* test comparisons support the Follow Both Model as the best explanation of liability judgments among U.S. laypeople. Specifically, ratings in the standard–accept condition were significantly higher than those in all other conditions. Ratings in the standard–reject condition were significantly lower than those in both nonstandard conditions. Ratings in the nonstandard–accept and nonstandard–reject conditions did not significantly differ.

For the third comparison, a TOST procedure indicated significant equivalence (*t* = −4.90; 90% CI, −0.1 to 0.36; *P* < 0.001), supporting the plausibility of the original null hypothesis that no difference exists between these 2 groups.

### German Physicians

For German physicians (41.9% female), as for the U.S. sample, the 2-way ANOVA revealed a significant effect of the physician’s decision (accept vs. reject; *F*(1, 244) = 6.64, *P* = 0.01) and no significant effect for the recommendation (standard vs. nonstandard; *F*(1, 244) = 1.29, *P* = 0.26). However, in this sample, there was no significant effect of the recommendation × decision interaction (*F*(1, 244) = 1.43, *P* = 0.23).

Mean reasonableness ratings were highest in the nonstandard–accept (mean, 60.07; SD, 1.39) and standard–accept (mean, 6.03; SD, 1.34) conditions, followed by nonstandard–reject (mean, 5.79; SD, 1.4) and standard–reject (mean, 5.31; SD, 1.68) (Fig. 4). Pairwise *t* test comparisons showed that ratings in the standard–accept condition were significantly higher than in the standard–reject condition but did not significantly differ from those in the nonstandard–accept and nonstandard–reject conditions. Ratings in the standard–reject condition were significantly lower than those in the nonstandard–accept condition yet were not significantly different from ratings in the nonstandard–reject condition. Ratings for nonstandard–accept and nonstandard–reject conditions did not significantly differ.

Taken together with the ANOVA results, these comparisons support the Follow Both Model. As for the U.S. sample, the TOST analysis indicated significant equivalence between nonstandard–accept and nonstandard–reject conditions (*t* = −1.76; 90% CI, –0.1 to 0.36; *P* = 0.04). For this sample, the significant TOST equivalence between the standard–accept and the nonstandard–accept conditions is particularly noteworthy (*t* = 2.6; 90% CI, −0.38 to 0.44; *P* = 0.005). Overall, German physicians seemed to place comparatively more weight on AI recommendations.

### German Laypeople

For German laypeople (52.58% female), similarly to U.S. laypeople, the 2-way ANOVA detected a significant effect of decision (accept vs. reject; *F*(1, 1354) = 4.38, *P* = 0.04) and of recommendation × decision interaction (*F*(1, 1354) = 13.96, *P* < 0.001). There was no significant effect of recommendation (standard vs. nonstandard; *F*(1, 1354) = 2.91, *P* = 0.09).

The highest reasonableness ratings were observed in the standard–accept condition (mean, 5.24; SD, 1.56), followed by nonstandard–reject (mean, 4.94; SD, 1.62), nonstandard–accept (mean, 4.80; SD, 1.64), and standard–reject (mean, 4.73; SD, 1.64) (Fig. 5). Pairwise *t* test comparisons revealed that ratings in standard–accept were significantly higher than in all other conditions. Ratings in standard–reject were not significantly different from those in nonstandard–reject or nonstandard–accept. Ratings in nonstandard–reject were also not significantly different from those in nonstandard–accept.

TOST procedures indicated statistical equivalence between nonstandard–reject and nonstandard–accept (*t* = 5.35; 90% CI, −0.35 to 0.06; *P* < 0.001) and between nonstandard–accept and standard–reject (*t* = −5.7; 90% CI −0.14 to 0.29; *P* < 0.001).

Although the overall pattern among German laypeople mirrored that of the U.S. cohort, differences emerged. In this sample, the Follow Both Model again best accounted for liability judgments. However, German lay participants appeared more sensitive to whether AI recommendations aligned with standard care.

### Pooled Dataset

Data from all 3 samples were aggregated to conduct a pooled analysis of differences in reasonableness judgments across groups.

We first examined baseline differences in perceived reasonableness across samples, regardless of condition. Regression analysis showed that German physicians rated decisions as more reasonable than did U.S. laypeople ($\beta_{1}$= 0.67; 95% CI, 0.45–0.89; *P* < 0.001; Supplemental Table 7; Supplemental Fig. 1), suggesting greater deference to the fictional physician’s choices. In contrast, German laypeople provided lower reasonableness ratings than did U.S. laypeople and German physicians ($\beta_{2}$= –0.19; 95% CI, –0.32 to –0.07; *P* = 0.002; Supplemental Table 7; Supplemental Fig. 1).

We then examined whether differences in response to the physician’s acceptance or rejection of AI advice varied by group. When averaged across conditions, decisions to reject AI recommendations were generally rated as less reasonable than decisions to accept them ($\beta_{1}$ = −0.44; 95% CI, –0.62 to –0.26; *P* < 0.001; Supplemental Table 8; Supplemental Fig. 2). However, this effect was contingent on whether the AI recommendation aligned with standard care (Supplemental Table 10) and differed by group. U.S. laypeople and German physicians responded similarly, whereas German laypeople showed marginally lower deference to AI-generated recommendations ($\beta_{4}$ = 0.25; 95% CI, 0–0.5; *P* = 0.05; Supplemental Table 8; Supplemental Fig. 2). Beyond this interaction, no other between-group differences in the effects of accepting versus rejecting AI advice reached statistical significance (Supplemental Figs. 3–4; Supplemental Tables 9–10).

## DISCUSSION

Despite the rapid shifts in public perceptions of AI, we replicate the findings of Tobia et al. (7), supporting the intertemporal robustness of that model of judgment. The experiment indicated that both factors—AI-recommended rather than AI-nonrecommended, and standard care rather than nonstandard care—increased participant ratings of reasonableness. The Follow Both Model best explains liability judgments among U.S. laypeople, who rated physicians more favorably when they accepted AI recommendations aligned with standard care. When AI recommended nonstandard care, there was no significant difference between accepting and rejecting the advice. These results suggest that U.S. tort law does not discourage physicians from following nonstandard AI advice and preserves clinical discretion in such cases.

We also extended that study to medical experts, finding that the same Follow Both Model best characterizes the judgments of German physicians. Similar to U.S. laypeople, German physicians rated the fictional physician’s decision more favorably when it was aligned with standard care. When AI recommended nonstandard care, there was no meaningful difference between accepting and rejecting the advice. However, physicians were comparatively more influenced by AI recommendations.

Two notable differences emerged between laypeople and German physicians. First, German physicians were generally more lenient in evaluating the fictional physician’s decisions. Second, unlike lay participants, who rated acceptance of standard advice as more reasonable than acceptance of nonstandard advice, German physicians rated both types of acceptance similarly. This suggests that German physicians view acceptance of AI advice, whether standard or nonstandard, as equally reasonable. Overall, these findings suggest that the German tort system does not discourage physicians from accepting AI-generated advice and, if anything, might encourage them to accept it.

When U.S. laypeople are compared with German physicians, observed differences might be attributable to broader cultural distinctions between the United States and Germany. We tested this hypothesis with the inclusion of German laypeople. German lay participants displayed a notably more skeptical attitude toward medical professionals than did German physicians. As with the other groups, the Follow Both Model best characterized their responses, although the German laypeople were comparatively more responsive to the standard of care.

Some limitations warrant consideration. First, we use a factorial vignette experiment to cleanly identify causal effects on judgments of physician reasonableness, but this design inevitably simplifies the complexities of real-world trials. Yet, in the absence of sufficient real-world negligence cases involving medical AI, controlled simulations remain a valuable tool for exploring liability judgments. Second, our findings are derived from a single stylized oncology vignette and should therefore be interpreted in light of that specific scenario. The case was deliberately retained from prior work to enable direct replication, but this choice limits generalizability. Judgments may differ in clinical settings with different stakes, time pressures, disease severity, familiarity with pharmacologic agents involved, or therapeutic discretion. Moreover, whereas the physician sample was drawn from academic centers (including a comprehensive oncologic center), it may not fully reflect the level of subspecialty expertise and forensic experience typically associated with court-appointed medical experts in malpractice proceedings. Third, all liability models we examined assume that harm has occurred. In practice, liability risk depends on both the likelihood of being found liable given harm and the probability of harm itself. Although our design addressed the former, holding the occurrence of harm constant across conditions, it cannot assess the latter, which is context-dependent and subject to physicians’ own assessment. Fourth, our findings pertain to physicians’ decisions to follow recommendations by medical AI tools already adopted by hospitals. They do not address whether, from a liability perspective, hospitals should adopt such tools in the first place: a question for future research.

From a clinical standpoint, our findings have important implications for physicians working in hospitals that adopt medical AI tools. Although physicians do not make treatment decisions by mechanically tracking anticipated liability perceptions, malpractice norms may still affect their decisions indirectly through hospital policies, professional guidance, and insurer or institutional risk-management practices. To minimize liability risk, physicians are strongly incentivized to follow standard-care AI recommendations. Overruling such advice would require a firm belief that nonstandard care offers a significantly better outcome. By contrast, when AI provides nonstandard recommendations, the legal framework allows for greater clinical discretion. Finally, the differing perspectives of German laypeople and physicians may also reflect a gap between expert judgment and patient expectations—an insight that could inform the design of more effective informed consent practices.

## CONCLUSION

In samples drawn from jury-based and expert-based legal systems, we find consistent evidence that physicians who reject medical AI advice are judged less favorably than those who accept it. When AI recommendations align with standard care, acceptance is associated with higher perceived reasonableness; when AI recommends nonstandard care, accepting and rejecting the advice are judged as equally reasonable. Together, our findings suggest that malpractice liability regimes do not necessarily pose a barrier to the use of AI in precision medicine.

## DISCLOSURE

This research was funded through the discretionary research funds provided by ETH Zurich to Prof. Alexander Stremitzer. No other potential conflict of interest relevant to this article was reported.

## Notes

KEY POINTS **QUESTION:** How can physicians minimize malpractice liability when deciding to accept or reject medical AI recommendations? **PERTINENT FINDINGS:** In this randomized vignette experiment including 248 German physicians, 1202 U.S. adults, and 1358 German adults, physicians who accepted AI advice consistent with standard care were judged more reasonable than those who rejected it. When AI recommended nonstandard care, accepting and rejecting the advice were judged as equally reasonable. **IMPLICATIONS FOR PATIENT CARE:** Across jury-based and expert-based legal systems, following AI advice does not appear to increase malpractice risk, suggesting that liability concerns do not necessarily undermine responsible AI integration in clinical practice.
